# Differences in Reproductive Performance Between Shiqi and Dabao Pigeons: Insights from Multi-Omics Analysis

**DOI:** 10.3390/genes17091049

**Published:** 2026-08-30

**Authors:** Xingyu Tang, Chenbo Zhao, Jinquan Xi, Tieshan Xu, Tiantian Gu, Jindong Ren, Shihong Liu, Lihong Gu, Lizhi Lu

**Affiliations:** 1 Key Laboratory of Livestock and Poultry Resources (Poultry) Evaluation and Utilization, Ministry of Agriculture and Rural Affairs, Institute of Animal Husbandry and Veterinary Science, Zhejiang Academy of Agricultural Sciences, Hangzhou 310021, China; 2 Haikou Coconut Pigeon Food Breeding Co., Ltd., Haikou 571133, China; 3 Tropical Crops Genetic Resources Institute, Chinese Academy of Tropical Agricultural Sciences, Haikou 571101, China; xutieshan760412@163.com; 4 Institute of Animal Science and Veterinary Medicine, Hainan Academy of Agricultural Sciences, Haikou 571199, China

**Keywords:** pigeon, ovary, follicle development, transcriptomics, metabolomics

## Abstract

**Background/Objectives:** Shiqi and Dabao pigeons are two Chinese meat-pigeon breeds with distinct breeding backgrounds and production-related phenotypes, but whether these differences extend to ovarian physiology and molecular profiles remains unclear. This study compared ovarian physiological traits and multi-omics profiles between the breeds. **Methods:** Seven-month-old reproductively mature females were studied. Flock-level laying and fertilization records were obtained from 456 breeding pairs per breed and recorded every 2 days. Serum reproductive hormones were measured in 10 females per breed. Follicle numbers, atresia, and mature follicle diameter were evaluated in three females per breed. Ovarian samples from six females per breed were analyzed by paired-end RNA sequencing and untargeted LC–MS/MS metabolomics in positive- and negative-ion modes. **Results:** Dabao pigeons had higher serum progesterone concentrations than Shiqi pigeons (1.35 ± 0.49 vs. 0.32 ± 0.19 ng/mL, *p* < 0.001) and larger mature follicles (2.25 ± 0.31 vs. 1.08 ± 0.56 mm, *p* = 0.034), whereas follicle numbers were not significantly different. Transcriptomic analysis identified 761 DEGs (FC > 2 or < 0.5; adjusted *p* < 0.05), mainly involving extracellular-matrix, cholesterol-, estrogen-, PI3K–Akt-, and TGF-β-related processes. Metabolomic analysis identified five differential metabolites (VIP > 1, FC > 2 or < 0.5; adjusted *p* < 0.05), including three taurine-conjugated bile acids. Integrated analysis revealed exploratory gene–metabolite associations related to lipid metabolism, follicular signaling, and extracellular-matrix remodeling. **Conclusions:** Shiqi and Dabao pigeons differed mainly in ovarian physiological and molecular characteristics, whereas short-term flock-level reproductive records were broadly similar. Metabolomic differences were limited but concentrated in bile-acid- and lipid-related features.

## 1. Introduction

Meat pigeon production is an important component of China’s specialty poultry industry, and its sustainable development depends on improved germplasm resources and efficient breeding systems [1]. Compared with major poultry species, however, the meat pigeon industry remains relatively underdeveloped in terms of breeding stock selection and genetic-resource utilization. Relatively low reproductive efficiency has therefore become an important constraint on productivity and industrial development [2].

Domestic pigeons have distinctive reproductive characteristics. Breeding pigeons generally form stable pairs and typically produce two eggs per clutch, with an egg-laying cycle of approximately 8–12 days; the overall reproductive cycle is longer when mating, incubation, and parental care are included [3,4]. Under commercial management, laying may begin at approximately 6 months of age. Meat-type breeding pigeons are generally maintained as male–female pairs and reproduce through natural mating and parental incubation, although reproductive performance may vary seasonally. In addition, reproductive maturation is influenced by factors such as photoperiod [5]. These characteristics make reproductive efficiency closely dependent on endocrine status, ovarian function, and breeding management.

Shiqi and Dabao pigeons are two Chinese meat-pigeon breeds with distinct breeding backgrounds and growth- and carcass-related phenotypes. Shiqi pigeons originated in Zhongshan, Guangdong Province, and were developed through long-term selection involving local pigeons and introduced meat-pigeon breeds, whereas Dabao pigeons were developed through a five-year selection program in Guangdong Province in the 1990s [6,7]. Previous studies have reported differences between these breeds in growth and production-related traits, with Dabao pigeons showing advantages in growth rate, body weight, and several body-size traits, whereas Shiqi pigeons showed a higher dressing percentage [8]. These differences provide a biological basis for investigating whether the two breeds also differ in ovarian physiology.

Pigeon reproduction is regulated by the hypothalamic–pituitary–gonadal axis, which coordinates gonadotropin secretion [9], ovarian steroidogenesis, follicular development [10], and ovulation [11,12]. Previous studies have linked reproductive variation in poultry to changes in ovarian gene expression and metabolism, particularly in steroidogenesis, follicular development, and lipid metabolism [13,14,15]. Integrated transcriptomic and metabolomic analyses have further revealed coordinated molecular changes associated with reproductive traits [16,17]. However, whether such multi-level differences occur between Shiqi and Dabao pigeons remains unclear. Integrating omics data with reproductive hormone analysis and ovarian histology may therefore help characterize breed-associated ovarian variation.

We hypothesized that the distinct breeding backgrounds and growth- and carcass-related phenotypes of Shiqi and Dabao pigeons are associated with differences in ovarian physiological status and transcriptomic and metabolic profiles. Accordingly, we compared serum reproductive hormones, ovarian histomorphology, transcriptomic profiles, and metabolomic profiles between the two breeds and integrated these datasets to identify candidate molecular features associated with breed-related ovarian variation.

## 2. Materials and Methods

### 2.1. Animal Ethics Statement

All animal procedures were approved by the Animal Ethics Committee of Zhejiang Academy of Agricultural Sciences (approval No. 26ZALAS18; date of approval: 7 April 2026) and were conducted in accordance with institutional and national guidelines for the care and use of laboratory animals. All blood collection, euthanasia, and ovarian tissue sampling were conducted after ethical approval was obtained on 7 April 2026. The study is reported in accordance with the ARRIVE guidelines.

### 2.2. Study Design

This study used a cross-sectional comparative design to evaluate ovarian physiological and molecular differences between 7-month-old Shiqi and Dabao breeding pigeons. Commercial production records were obtained from separate breeding populations comprising 456 breeding pairs of each breed. Each breeding pair consisted of one male and one female housed in an individual cage. These production populations were distinct from the pigeons used for physiological and multi-omics analyses. Serum reproductive hormones were measured in 10 females per breed. For ovarian histomorphological analysis, three females were randomly selected from each breed, and three representative H&E-stained sections from each pigeon were analyzed. Measurements from the three sections were averaged to obtain one animal-level value, with the individual pigeon considered the experimental unit (*n* = 3 per breed). Ovarian samples from six females per breed were used for transcriptomic and metabolomic analyses, with the same individuals used for both omics platforms.

### 2.3. Experimental Animals and Sample Collection

Ten healthy 7-month-old breeding pigeons from each breed, Shiqi and Dabao, with comparable body weights, were obtained from Haikou Coconut Pigeon Food Breeding Co., Ltd., Haikou, Hainan, China. Seven-month-old pigeons were selected because they had reached reproductive maturity and were being maintained as active breeders under routine commercial conditions. Selecting females of the same age and comparable reproductive status was intended to minimize age-related variation in ovarian physiology between the two breeds. All pigeons were maintained under the same routine commercial breeding conditions, with free access to feed and clean drinking water, and were managed according to the standard husbandry practices of Haikou Coconut Pigeon Food Breeding Co., Ltd. Serum and ovarian tissue samples were collected from all pigeons. A portion of each ovary was fixed for histological examination, whereas serum and the remaining ovarian tissues were rapidly frozen in liquid nitrogen and stored at −80 °C for subsequent biochemical assays and multi-omics analyses. Blood samples were collected from the wing vein before euthanasia. Pigeons were anesthetized by intravenous injection of sodium pentobarbital and subsequently euthanized by exsanguination. Ovarian tissues were collected immediately after euthanasia.

### 2.4. Experimental Procedures

#### 2.4.1. Egg-Laying and Fertilization Records

Production records were obtained from commercial breeding populations comprising 456 breeding pairs of Shiqi pigeons and 456 breeding pairs of Dabao pigeons. Each breeding pair consisted of one male and one female housed separately in an individual cage. Fertilization occurred through natural mating without artificial insemination, and eggs were incubated naturally by the parental breeding pairs. Records were collected every 2 days over a 30-day observation period spanning June and July 2026. At each recording time, the numbers of eggs laid, fertilized eggs, infertile eggs, and broken or otherwise unevaluable eggs were recorded and summed across all cages within each breed. Fertilization rate was calculated as the number of fertilized eggs divided by the total number of eggs examined and expressed as a percentage. Because only aggregated flock-level records were retained and cage-level observations were unavailable as independent replicates, these data were summarized descriptively and were not subjected to breed-level inferential statistical testing.

#### 2.4.2. Hematoxylin and Eosin Staining

Formalin-fixed ovarian tissues were dehydrated through a graded ethanol series, cleared in xylene, embedded in paraffin, and cut into 5 μm sections. The sections were stained with hematoxylin and eosin (H&E) and observed under a light microscope. Follicles were classified as primordial, growing, or mature follicles according to their morphology. Follicle numbers and mature follicle diameter were measured using ImageJ software (version 1.54g, National Institutes of Health, Bethesda, MD, USA). Representative sections with clear tissue structure were selected for quantitative analysis. Preparation of H&E-stained sections was performed by Hangzhou Zhiqi Biotechnology Co., Ltd. (Hangzhou, China), Three pigeons per breed were randomly selected for quantitative histomorphological analysis, and three representative H&E-stained sections were evaluated from each pigeon. Follicle counts and mature follicle diameters from the three sections were averaged to obtain one animal-level value for each parameter. Thus, the individual pigeon, rather than the histological section, represented the experimental unit (*n* = 3 per breed).

#### 2.4.3. Measurement of Serum Reproductive Hormones

Blood samples were collected from the wing vein during the afternoon before euthanasia, with all birds sampled within the same general time period to reduce potential variation associated with sampling time. Serum concentrations of follicle-stimulating hormone (FSH), luteinizing hormone (LH), progesterone (P4), and estradiol (E2) were determined using commercial ELISA kits (Beijing Huaying Biotechnology Research Institute, Beijing, China; Cat. Nos. HY-C0001, HY-C0002, HY-C0006, and HY-C0007, respectively) according to the manufacturer’s instructions. Absorbance was measured at 450 nm, and hormone concentrations were calculated from the corresponding calibration curves. Because these kits were not specifically developed for pigeons, the calibration curves used for hormone quantification are provided in Supplementary Figure S1.

#### 2.4.4. Ovarian Transcriptome Sequencing and Analysis

Ovarian tissue samples from Shiqi and Dabao pigeons were submitted to Personalbio Technology Co., Ltd. (Shanghai, China), for RNA extraction, library construction, and transcriptome sequencing. Total RNA was extracted from frozen ovarian tissues, and RNA concentration, purity, and integrity were assessed before library preparation. Qualified RNA samples were used for mRNA library construction. Briefly, poly(A)-containing mRNA was enriched using Oligo(dT) magnetic beads, fragmented to approximately 300 bp, and reverse-transcribed into cDNA. After second-strand synthesis, end repair, adaptor ligation, PCR amplification, and size selection, the libraries were checked using an Agilent 2100 Bioanalyzer (Agilent Technologies, Waldbronn, Germany). Indexed libraries were pooled and sequenced on an Illumina NovaSeq platform using a paired-end 2 × 150 bp strategy.

Raw reads were filtered to remove 3′ adaptor sequences and reads with an average quality score below Q20. The resulting clean reads were aligned to the pigeon reference genome GCF_036013475.1_bColLiv1.pat.W.v2_genomic.fna. Gene-level read counts were obtained based on the genome annotation, and fragments per kilobase of transcript per million mapped fragments (FPKM) values were calculated for expression-level visualization. Sequencing quality and sample consistency were evaluated using Q20, Q30, read distribution, sequencing saturation, gene-body coverage, inter-sample correlation, and PCA. PCA and inter-sample correlation analyses were used to identify potential outliers. All 12 libraries met the sequencing quality-control requirements, and no samples were excluded from downstream analysis. All libraries were prepared and sequenced within the same experimental batch; therefore, no technical batch covariate was included in the differential-expression model.

Differential expression analysis between the ovaries of Shiqi and Dabao pigeons was performed using DESeq2 based on gene read counts. *p* values were adjusted for multiple testing using the Benjamini–Hochberg procedure. Genes with FC > 2 or FC < 0.5 and an adjusted *p* value < 0.05 were considered differentially expressed genes (DEGs). Hierarchical clustering of DEGs was performed using the pheatmap package in R with Euclidean distance and complete linkage clustering. Gene Ontology (GO) and Kyoto Encyclopedia of Genes and Genomes (KEGG) enrichment analyses were performed using the annotated gene set as the background to identify biological functions and pathways associated with the DEGs. All downstream DEG-based analyses were performed using the FDR-adjusted DEG set defined above.

#### 2.4.5. Ovarian Metabolomic Profiling and Analysis

Ovarian tissue samples were submitted to Personalbio Technology Co., Ltd. (Shanghai, China) for untargeted liquid chromatography–tandem mass spectrometry (LC–MS/MS) metabolomic analysis. Frozen ovarian tissues were extracted using a pre-cooled organic extraction solution. After vortexing, ultrasonic extraction, protein precipitation, and centrifugation, the supernatants were collected for LC–MS/MS analysis. Quality control (QC) samples were prepared by pooling equal aliquots from all individual samples and were analyzed throughout the run to monitor platform stability.

Raw metabolomic data were processed for peak detection, alignment, normalization, and metabolite annotation. Metabolites were annotated based on retention time, accurate mass, MS/MS fragmentation spectra, and collision energy information in comparison with reference databases. A mass error threshold of less than 10 ppm was used, and metabolite annotation results were manually checked. QC chromatograms, QC sample correlation, relative standard deviations (RSDs), and PCA were used to evaluate data quality. All metabolomic samples met the quality-control requirements and were retained for downstream statistical analysis.

For group comparison, principal component analysis (PCA) was first used as an unsupervised approach to evaluate overall sample distribution and potential outliers. Orthogonal partial least squares discriminant analysis (OPLS-DA) was subsequently performed separately for the positive- and negative-ion datasets to characterize metabolic differences between Shiqi and Dabao pigeon ovaries. Model performance was evaluated using R^2^Y and Q^2^ values, and potential model overfitting was assessed by permutation testing. Variable importance in projection (VIP) scores were obtained from the OPLS-DA models, with VIP > 1 indicating an above-average contribution to group discrimination. Univariate *p* values were adjusted for multiple comparisons using the Benjamini–Hochberg procedure. Metabolites with VIP > 1, FC > 2 or FC < 0.5, and an adjusted *p* value < 0.05 were considered differentially abundant. KEGG pathway analysis was subsequently performed using the metabolites that met these revised criteria.

#### 2.4.6. Integrated Transcriptomic and Metabolomic Analysis

FDR-significant DEGs annotated to predefined functional categories related to ovarian steroidogenesis, lipid metabolism, follicular signaling, and extracellular-matrix remodeling were selected for focused gene–metabolite correlation analysis with the FDR-significant differential metabolites. Gene expression levels and metabolite abundances were matched according to the corresponding ovarian samples. Spearman correlation analysis was used to evaluate gene–metabolite associations. *p* values from the correlation analyses were adjusted for multiple testing using the Benjamini–Hochberg procedure. Correlation heatmaps and gene–metabolite networks were used to visualize significant associations. These analyses were considered exploratory and were not used to infer direct causal or regulatory relationships.

### 2.5. Statistical Analysis of Data

Statistical analyses of serum reproductive hormone concentrations and ovarian histomorphological measurements were performed using SPSS 26.0. For serum hormone measurements, the individual pigeon was considered the experimental unit (*n* = 10 per breed). For histomorphological measurements, the individual pigeon was also considered the experimental unit (*n* = 3 per breed); measurements from three H&E-stained sections of each pigeon were averaged before statistical analysis. Differences between Shiqi and Dabao pigeons were evaluated using independent-samples t-tests. Data are presented as mean ± SD, and *p* < 0.05 was considered statistically significant. Egg-laying and fertilization records were available only as repeated flock-level aggregates for each breed and therefore lacked independent cage-level biological replicates. Consequently, these production records were summarized descriptively and were not subjected to breed-level inferential statistical testing.

## 3. Results

### 3.1. Laying and Fertilization Records of 7-Month-Old Shiqi and Dabao Pigeons

As shown in Table 1, flock-level egg-laying and fertilization records were broadly comparable between the Shiqi and Dabao commercial breeding populations. A total of 701 eggs were recorded from the 456 Shiqi breeding pairs and 704 eggs from the 456 Dabao breeding pairs. The corresponding numbers of fertilized eggs were 495 and 509, respectively. Because only aggregated flock-level records were available, these data were interpreted descriptively rather than as independent breed-level biological replicates.

### 3.2. Serum Reproductive Hormone Profiles in Shiqi and Dabao Pigeons

As shown in Table 2, serum LH and FSH concentrations were numerically higher in Shiqi pigeons than in Dabao pigeons, although neither difference reached statistical significance (LH: 7.44 ± 0.32 vs. 6.70 ± 1.05 IU/L, *p* = 0.102; FSH: 5.66 ± 0.44 vs. 5.00 ± 0.87 IU/L, *p* = 0.097). In contrast, serum progesterone concentration was significantly higher in Dabao pigeons (*p* < 0.001).

### 3.3. Ovarian Histology in Shiqi and Dabao Pigeons

**Figure 1:**
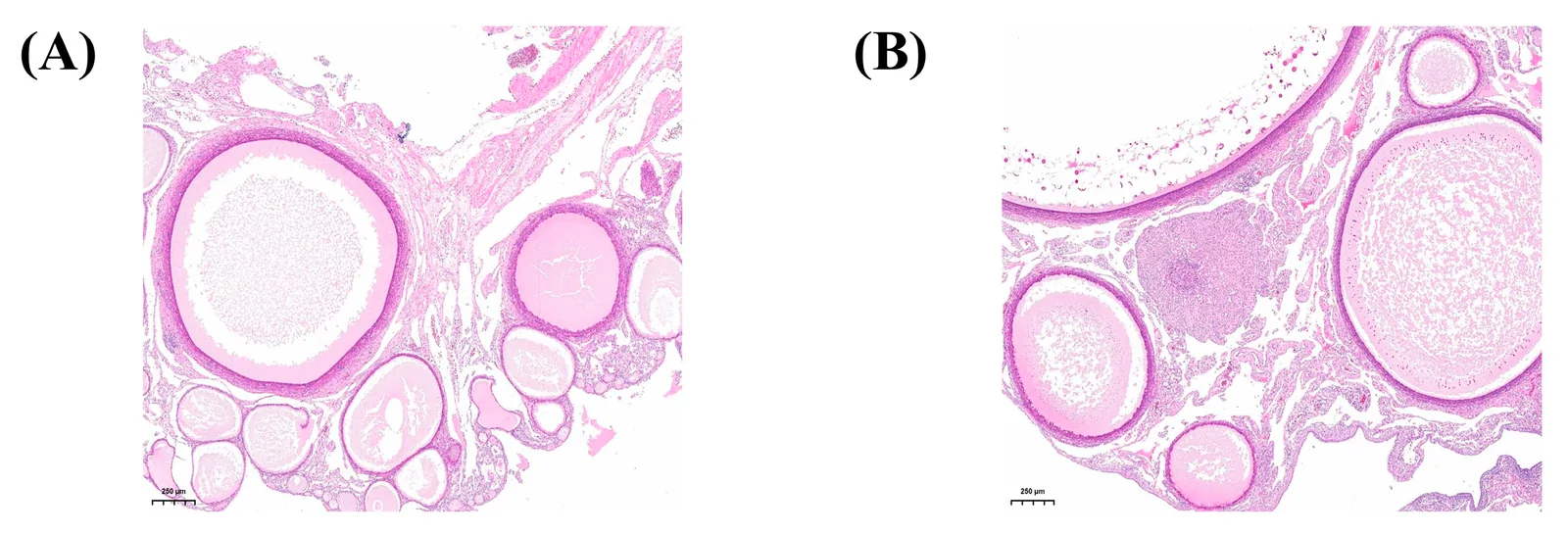
Histological characteristics of ovaries from Shiqi and Dabao pigeons. Representative hematoxylin and eosin-stained ovarian sections from Shiqi pigeons (**A**) and Dabao pigeons (**B**). Scale bars = 250 μm.

As shown in Figure 1, ovarian sections from both Shiqi and Dabao pigeons contained follicles at multiple developmental stages, including primordial follicles, growing follicles, and mature follicles.

Quantitative analysis of H&E-stained ovarian sections (Table 3) showed no significant differences between the two breeds in the number of primordial follicles, growing follicles, or mature follicles, nor in the proportion of atretic follicles (*p* > 0.05). In contrast, the mean diameter of mature follicles was significantly larger in Dabao pigeons than in Shiqi pigeons (*p* < 0.05). These results suggest that the two breeds were similar in overall follicular composition, but differed in the development of mature follicles.

### 3.4. Ovarian Transcriptomic Analysis of Shiqi and Dabao Pigeons

**Figure 2:**
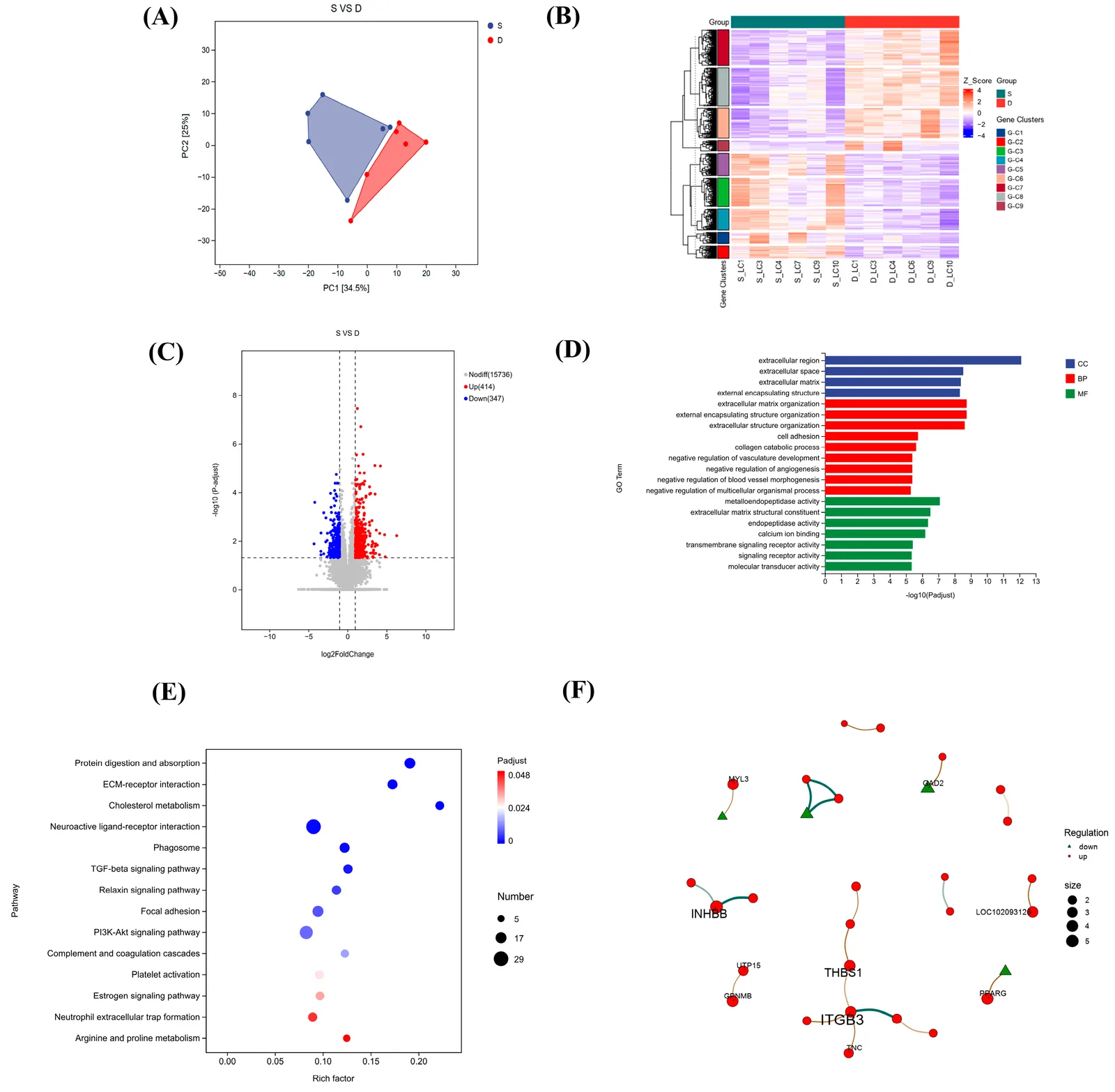
Transcriptomic analysis of ovaries from Shiqi and Dabao pigeons. (**A**) Principal component analysis score plot. (**B**) Hierarchical clustering. (**C**) Volcano plot of differentially expressed genes. (**D**) Gene Ontology enrichment. (**E**) Kyoto Encyclopedia of Genes and Genomes pathway enrichment. (**F**) Protein–protein interaction network.

Transcriptomic analysis showed distinct separation between the ovarian samples of Shiqi and Dabao pigeons. Principal component analysis and hierarchical clustering showed separation between the two breeds, with samples within each group displaying relatively consistent expression patterns (Figure 2A,B). A total of 761 differentially expressed genes (DEGs) were identified using the criteria of FC > 2 or FC < 0.5 and adjusted *p* < 0.05, including 414 upregulated and 347 downregulated genes in the S versus D comparison (Figure 2C). GO enrichment analysis indicated that these DEGs were mainly associated with extracellular matrix organization, cell adhesion, and signaling-related processes (Figure 2D). KEGG analysis further highlighted pathways including ECM–receptor interaction, cholesterol metabolism, estrogen signaling, TGF-β signaling, PI3K–Akt signaling, focal adhesion, and related metabolic and signaling pathways (Figure 2E). PPI analysis highlighted several highly connected candidate genes, including *ITGB3*, *THBS1*, *INHBB*, and *PPARG*, suggesting that the ovarian transcriptomic differences between the two breeds involve processes related to extracellular-matrix remodeling, lipid metabolism, and reproductive signaling (Figure 2F).

### 3.5. Ovarian Metabolomic Analysis of Shiqi and Dabao Pigeons

**Figure 3:**
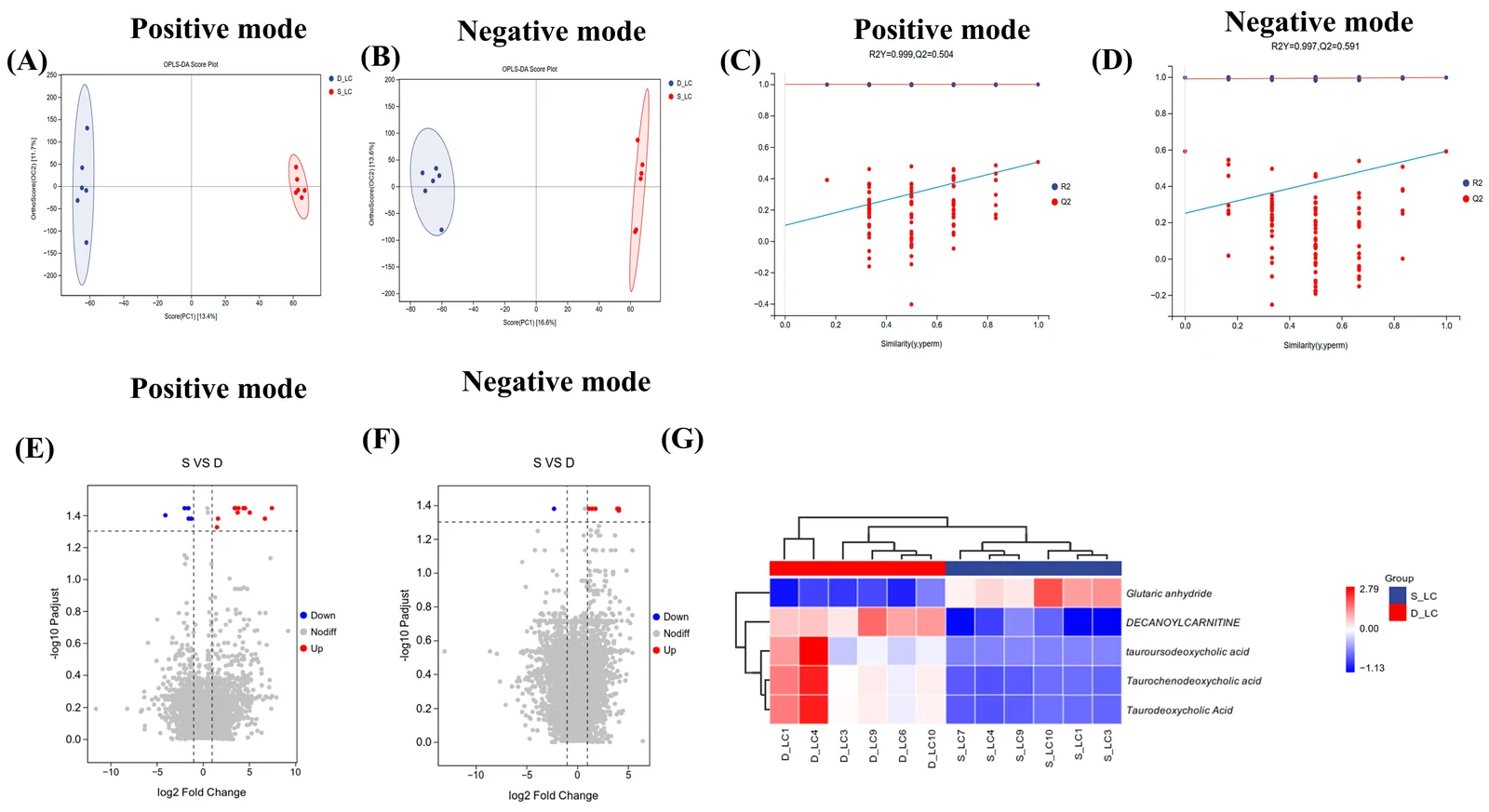
Metabolomic analysis of ovaries from Shiqi and Dabao pigeons. (**A**,**B**) Orthogonal partial least squares discriminant analysis (OPLS-DA) score plots in the two ionization modes. (**C**,**D**) Permutation tests for the corresponding OPLS-DA models. (**E**,**F**) Volcano plots based on FDR-adjusted differential-metabolite screening. (**G**) Hierarchical clustering heatmap of the five FDR-significant differential metabolites.

Metabolomic profiling showed separation between the ovarian samples of Shiqi and Dabao pigeons in the OPLS-DA models (Figure 3A,B). The positive-ion model yielded an R^2^Y of 0.999 and Q^2^ of 0.504, whereas the negative-ion model yielded an R^2^Y of 0.997 and Q^2^ of 0.591. The corresponding permutation tests produced Q^2^ intercepts of 0.19 and 0.15, respectively, indicating no obvious overfitting of the models (Figure 3C,D). Following Benjamini–Hochberg multiple-testing correction, five metabolites remained significantly different between the two breeds based on the revised criteria of VIP > 1, FC > 2 or FC < 0.5 and adjusted *p* < 0.05 (Figure 3E,F). Of these, taurodeoxycholic acid, taurochenodeoxycholic acid, tauroursodeoxycholic acid, and decanoylcarnitine showed higher abundances in Dabao pigeons, whereas glutaric anhydride showed a higher abundance in Shiqi pigeons. Hierarchical clustering of these five metabolites showed a clear breed-associated abundance pattern (Figure 3G). Notably, three of the five metabolites were taurine-conjugated bile acids, indicating that bile-acid-related metabolism represented a prominent feature of the FDR-significant metabolic differences between the two breeds. Pathway analysis of these metabolites highlighted primary bile acid biosynthesis; because only a limited number of metabolites remained significant after multiple-testing correction, this pathway-level interpretation was considered exploratory.

### 3.6. Correlation Analysis of Transcriptomic and Metabolomic Data

**Figure 4:**
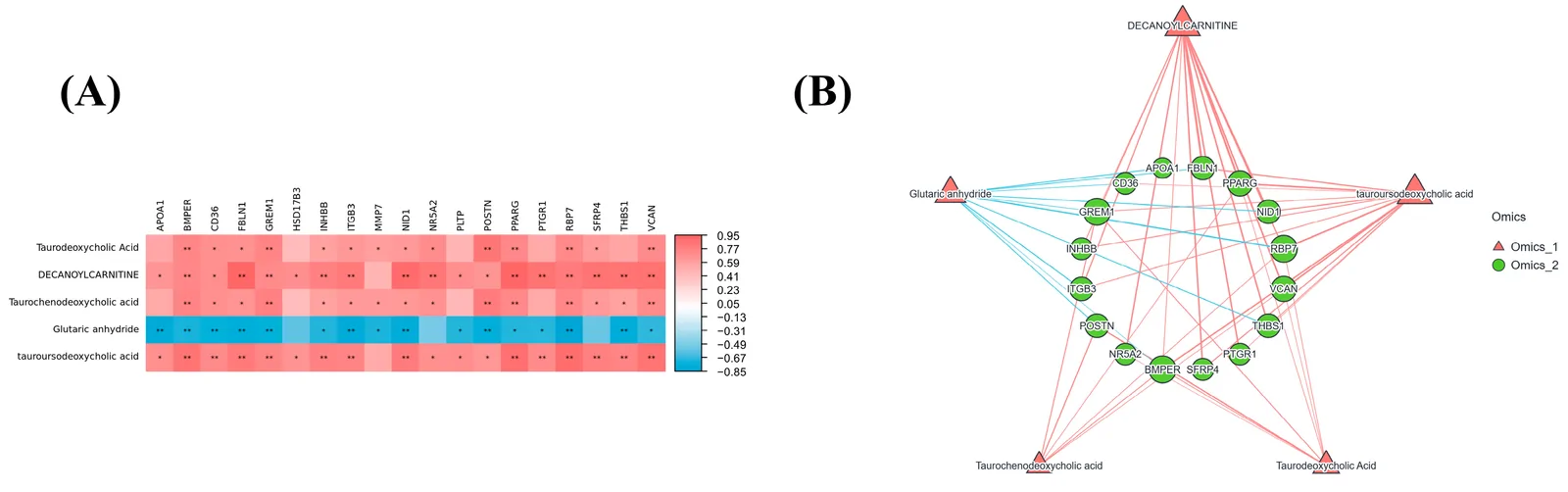
Integrated transcriptomic and metabolomic analysis of ovaries from Shiqi and Dabao pigeons. (**A**) Correlation heatmap between selected FDR-significant DEGs and the five FDR-significant differential metabolites. (**B**) Gene–metabolite correlation network showing selected significant associations. Red lines indicate positive correlations, whereas blue lines indicate negative correlations. Asterisks indicate statistical significance: * *p* < 0.05 and ** *p* < 0.01.

Integrated transcriptomic and metabolomic analysis identified associations between the five FDR-significant metabolites and selected ovarian DEGs related to lipid metabolism, endocrine signaling, follicular regulation, and extracellular-matrix remodeling (Figure 4A). Taurodeoxycholic acid, taurochenodeoxycholic acid, tauroursodeoxycholic acid, and decanoylcarnitine showed predominantly positive correlations with the selected DEGs, whereas glutaric anhydride generally showed inverse correlations. The correlation network further highlighted associations involving genes such as *PPARG*, *CD36*, *APOA1*, *NR5A2*, *INHBB*, *GREM1*, *ITGB3*, *THBS1*, *VCAN*, and other genes associated with ovarian metabolic or structural processes (Figure 4B). Together, these results indicate that the limited but robust metabolic differences identified after FDR correction were associated with breed-associated differences in ovarian transcriptional profiles, particularly those related to lipid metabolism, endocrine signaling, follicular regulation, and extracellular-matrix remodeling. However, these gene–metabolite associations are exploratory and do not establish direct regulatory or causal relationships.

## 4. Discussion

### 4.1. Ovarian Physiological Divergence and Reproductive Phenotypes

Progesterone is involved in terminal follicular maturation and ovulation, while mature follicle diameter reflects follicular growth and yolk deposition [18,19]. In this study, Dabao pigeons showed higher serum progesterone concentrations and larger mature follicles, whereas follicle numbers did not differ significantly between breeds. These findings suggest differences in ovarian physiological status and follicular maturation rather than superior reproductive performance, particularly because the measurements were obtained at a single sampling time.

Previous studies indicate that reproductive function depends not only on follicle number but also on follicular maturation, steroidogenic activity, and follicular quality [20,21,22]. Thus, the absence of significant differences in follicle number does not exclude physiological differences between breeds. Although LH and FSH concentrations were numerically higher in Shiqi pigeons (*p* = 0.102 and 0.097), these differences were not statistically significant. Because pituitary function was not directly examined, they should be interpreted only as possible differences in the endocrine environment of the HPG axis rather than evidence of altered pituitary function.

### 4.2. Ovarian Transcriptomic Divergence and Regulatory Remodeling

After Benjamini–Hochberg correction, 761 DEGs remained significant, indicating that a substantial number of ovarian transcriptomic differences between Shiqi and Dabao pigeons were retained after multiple-testing adjustment. Functional enrichment analysis highlighted ECM–receptor interaction, PI3K–Akt signaling, TGF-β signaling, focal adhesion, cholesterol metabolism, and estrogen signaling pathways. These pathways are involved in follicular development, extracellular-matrix remodeling, lipid metabolism, and ovarian signaling [23,24].

Several FDR-significant candidate genes, including *INHBB*, *PPARG*, *THBS1*, and *ITGB3*, were located within these functional networks. Previous studies have linked *INHBB* with follicular regulation [25], *PPARG* with lipid metabolism and granulosa-cell function [26], and *THBS1* and *ITGB3* with extracellular-matrix and vascular remodeling [27,28]. These findings support breed-associated differences in ovarian molecular state; however, the identified genes should be regarded as candidate markers rather than experimentally validated regulators.

### 4.3. Ovarian Metabolic Variation and Lipid-Related Features

After Benjamini–Hochberg correction, five metabolites remained significantly different between the two breeds, indicating a relatively focused metabolomic difference. Notably, taurodeoxycholic acid, taurochenodeoxycholic acid, and tauroursodeoxycholic acid, which are all taurine-conjugated bile acids, showed higher abundances in Dabao pigeons, whereas decanoylcarnitine was also increased and glutaric anhydride was decreased. Pathway analysis highlighted primary bile acid biosynthesis, suggesting that bile-acid-related metabolism represents a metabolic feature that distinguishes the two breeds [29].

Previous metabolomic studies in breeding pigeons have also linked reproductive status with alterations in lipid and energy metabolism [30]. Bile acids are downstream products of cholesterol metabolism and have been associated with ovarian function and reproductive performance in poultry [31]. Changes in bile-acid profiles, including taurine-conjugated bile acids, have also been linked to differences in lipid and cholesterol metabolism in laying hens [32]. Acylcarnitines such as decanoylcarnitine reflect fatty-acid utilization and mitochondrial lipid metabolism, and altered ovarian acylcarnitine profiles have been associated with altered ovarian functional status in hens [33]. Integrated transcriptomic and metabolomic studies in pigeons further support an association between reproductive physiology and coordinated lipid-related molecular changes [34]. These findings are therefore consistent with the cholesterol- and lipid-related signals observed in the present transcriptomic analysis. However, because only five metabolites remained significant after FDR correction and no independent targeted metabolomic validation was performed, these metabolic associations should be interpreted cautiously and require confirmation in larger or independent datasets.

### 4.4. Integrated Omics Associations and Ovarian Functional Divergence

The integrated analysis focused on the five FDR-significant metabolites and selected FDR-significant DEGs associated with ovarian steroidogenesis, lipid metabolism, follicular signaling, and extracellular-matrix remodeling. The three taurine-conjugated bile acids and decanoylcarnitine showed predominantly positive associations with genes including *PPARG*, *CD36*, *APOA1*, *NR5A2*, *INHBB*, and several extracellular-matrix-related genes, whereas glutaric anhydride generally showed inverse associations. These patterns suggest that breed-associated ovarian differences may involve concurrent changes in lipid/bile-acid-related metabolism and ovarian transcriptional programs. However, these correlations are exploratory and do not demonstrate direct functional or causal relationships between individual genes and metabolites.

A key contribution of this study is the integration of ovarian physiology, histomorphology, transcriptomics, and FDR-controlled metabolomics to characterize breed-associated ovarian differences in two commercially important meat-pigeon breeds.

### 4.5. Study Limitations

This study has several limitations. First, the laying and fertilization records covered a limited observation period and may not fully represent long-term reproductive performance. Second, ovarian physiological and molecular measurements were obtained at a single age and sampling time, and reproductive-stage variation may therefore have influenced the results. Third, although multiple-testing correction was applied, the selected DEGs, metabolites, and gene–metabolite associations were not independently validated by qRT-PCR or targeted metabolomic assays. These molecular findings should therefore be considered exploratory, and future studies should include stage-matched sampling and independent validation of key genes and metabolites.

## 5. Conclusions

Short-term flock-level reproductive records were broadly similar between Shiqi and Dabao pigeons, whereas Dabao pigeons showed higher serum progesterone concentrations and larger mature follicles. After multiple-testing correction, 761 DEGs and five differential metabolites were identified, with the latter mainly comprising taurine-conjugated bile acids and decanoylcarnitine. Integrated analysis revealed exploratory associations between these metabolites and ovarian genes related to lipid metabolism, follicular signaling, and extracellular-matrix remodeling. Overall, breed-associated differences were more evident in ovarian physiological and molecular characteristics than in short-term flock-level reproductive records.

## Figures and Tables

**Table 1:** Laying and fertilization records of 7-month-old Shiqi and Dabao pigeons.

Breed	Number of Breeding Pairs	Total Eggs Laid	Infertile Eggs	Fertilized Eggs	Broken Eggs	Fertilization Rate (%)
Shiqi pigeons	456	701	194	495	12	71.29
Dabao pigeons	456	704	176	509	19	71.74

Production records were obtained from 456 breeding pairs per breed, with one breeding pair housed per cage. Values represent aggregated flock-level records over the entire observation period. Cage-level records were not retained; therefore, these data were used for descriptive comparison and were not subjected to breed-level inferential statistical testing.

**Table 2:** Serum reproductive hormone profiles of Shiqi and Dabao pigeons.

Breed	LH (IU/L)	FSH (IU/L)	P4 (ng/mL)	E2 (pg/mL)
Shiqi pigeons	7.44 ± 0.32	5.66 ± 0.44	0.32 ± 0.19	29.62 ± 6.71
Dabao pigeons	6.70 ± 1.05	5.00 ± 0.87	1.35 ± 0.49	36.57 ± 16.92
*p* value	0.102	0.097	<0.001	0.333

Values are presented as mean ± SD, *n* = 10 per breed. Differences between breeds were analyzed using an independent-samples *t*-test. LH, luteinizing hormone; FSH, follicle-stimulating hormone; P4, progesterone; E2, estradiol.

**Table 3:** Quantitative histological analysis of ovarian follicles in Shiqi and Dabao pigeons.

Parameter	Shiqi Pigeons	Dabao Pigeons	*p* Value
Number of primordial follicles (per section)	55.00 ± 10.00	38.33 ± 7.64	0.083
Number of growing follicles (per section)	30.33 ± 11.93	26.33 ± 9.07	0.668
Number of mature follicles (per section)	4.00 ± 1.73	3.33 ± 1.53	0.643
Proportion of atretic follicles (%)	3.63 ± 0.32	4.96 ± 1.15	0.125
Mean diameter of mature follicles (mm)	1.08 ± 0.56	2.25 ± 0.31	0.034

Values are presented as mean ± SD, *n* = 3 pigeons per breed. For each pigeon, measurements from three histological sections were averaged before statistical analysis.

## Data Availability

The RNA-seq data generated in this study have been deposited in the NCBI Sequence Read Archive under BioProject accession number PRJNA1492591. The metabolomics data and phenotypic datasets supporting the findings of this study are provided in the Supplementary Materials and are available from the corresponding author upon reasonable request.

## Supplementary Materials

The following supporting information can be downloaded at: https://www.mdpi.com/article/10.3390/genes17091049/s1, Table S1: RNA-seq quality metrics for individual ovarian samples; Table S2: Differentially expressed genes identified between Shiqi and Dabao pigeon ovaries; Table S3: GO and KEGG enrichment results for DEGs; Table S4: Differential metabolites identified by untargeted LC–MS/MS; Table S5: KEGG enrichment results for differential metabolites; Table S6: Gene–metabolite correlation results; Supplementary File S1: ARRIVE checklist; Figure S1. Standard calibration curves used for serum reproductive hormone quantification.
