# Supplementary material for: Differences in Reproductive Performance Between Shiqi and Dabao Pigeons: Insights from Multi-Omics Analysis
Source: Genes (Basel). 2026 Aug 30;17(9):1049. doi: 10.3390/genes17091049 (PMC13606080; doi:10.3390/genes17091049)
Supplement: Supplementary file 1 [file genes-17-01049-s001.zip › Supplementary_Figure_S1.pdf]

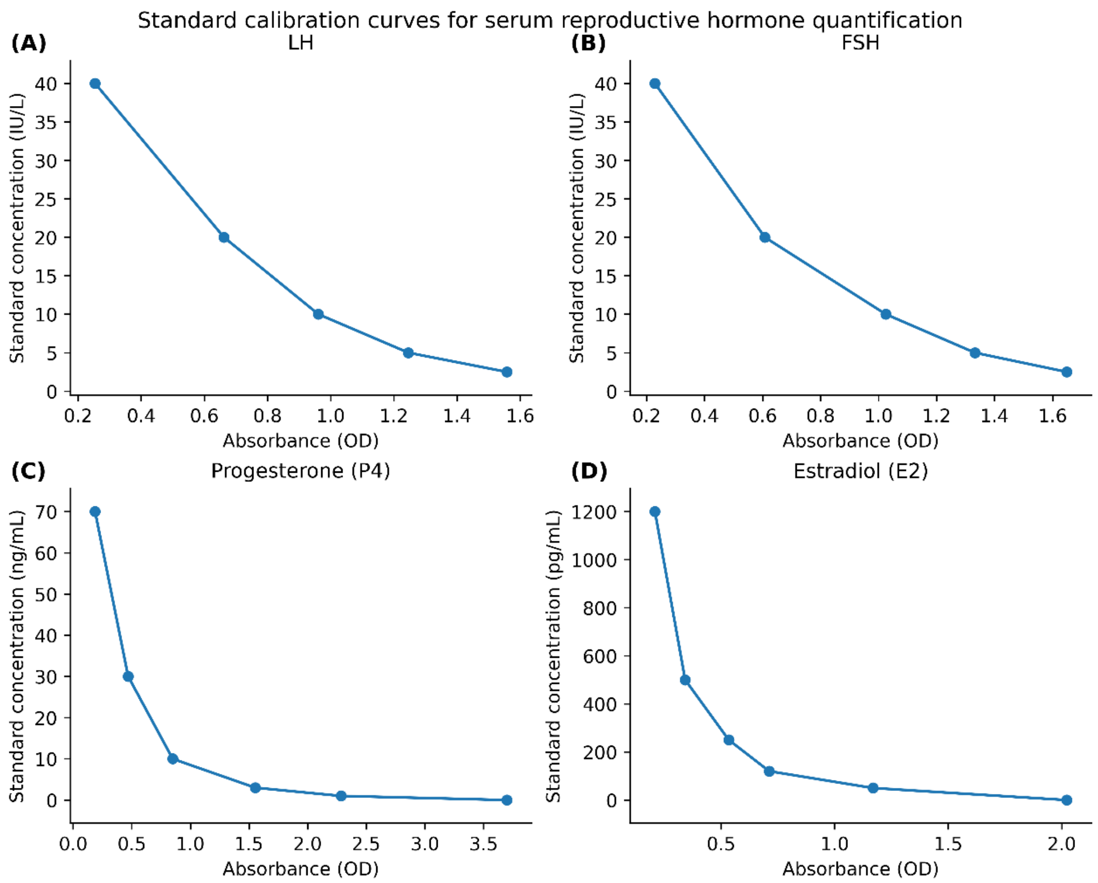

Figure S1. Standard calibration curves used for serum reproductive hormone quantification. (A) Luteinizing hormone (LH); (B) follicle-stimulating hormone (FSH); (C) progesterone (P4); and (D) estradiol (E2).
